# Biogenesis of Extracellular Vesicles (EVs) and the Potential Use of Embryo-Derived EVs in Medically Assisted Reproduction

**DOI:** 10.3390/ijms26010042

**Published:** 2024-12-24

**Authors:** Andreja Ovčar, Borut Kovačič

**Affiliations:** Department of Reproductive Medicine and Gynecological Endocrinology, University Medical Centre Maribor, 2000 Maribor, Slovenia; andreja.ovcar@ukc-mb.si

**Keywords:** extracellular vesicles, embryo culture medium, human embryo, biomarkers

## Abstract

Extracellular vesicles (EVs) are lipid bilayer-bound particles released from cells that cannot replicate on their own, play a crucial role in intercellular communication, and are implicated in various physiological and pathological processes. Within the domain of embryo culture media research, extensive studies have been conducted to evaluate embryo viability by analyzing spent culture medium. Advanced methodologies such as metabolomic profiling, proteomic and genomic analyses, transcriptomic profiling, non-coding RNA assessments, and oxidative status measurements have been employed to further understand the molecular characteristics of embryos and improve selection criteria for successful implantation. In the field of EVs, only a limited number of studies have been conducted on embryo-conditioned medium, indicating a significant gap in knowledge regarding the potential role of EVs in embryo development and implantation. Therefore, this review aims to evaluate current research findings on EVs enriched from animal and human embryo spent medium. By unraveling the potential link between embryo-derived EVs and embryo selection in clinical settings, such research might enhance embryo-selection methods in assisted reproductive technologies, eventually increasing the success rates of fertility treatments and advancing our understanding of mechanisms underlying successful embryo development and implantation in humans.

## 1. Introduction

Extracellular vesicles (EVs) are increasingly recognized as critical mediators of intercellular communication across various reproductive stages and are found abundantly in biological fluids like plasma, amniotic fluid, follicular fluid, and seminal fluid [1]. They carry a variety of bioactive cargo such as proteins, RNAs, microRNAs, and growth factors, with cargo depending on the cell of origin [2]. In the male reproductive system, EVs in seminal plasma, such as epididymosomes and prostasomes, facilitate key functions including sperm maturation, motility, and capacitation [3,4,5]. In the female reproductive tract, EVs from follicular and oviductal fluid play a vital role in oocyte maturation and embryo development. Follicular fluid EVs influence the proliferation of cumulus cells, follicular growth, and meiotic resumption in oocytes, processes that are regulated by cargo like miRNAs and proteins involved in MAPK pathways that impact granulosa cell proliferation and follicular growth [6,7]. In oviductal fluid, EVs support early embryo development by facilitating cellular communication in the preimplantation stage. Studies on uterine fluid-derived EVs have revealed their capacity to modulate endometrial receptivity [8,9]. Additionally, embryo-derived EVs influence endometrial cells to establish immune tolerance and promote vascular remodeling [10,11]. The ability of EVs to carry genetic material, such as miRNAs and DNA from the embryo itself, offers an exciting opportunity for their use as potential biomarkers in assisted reproductive technologies (ART) [12,13].

Therefore, spent culture medium (SCM)-derived EVs, carrying miRNA and DNA reflective of the embryo’s genomic status, hold the potential to replace preimplantation genetic testing (PGT) for assessing aneuploidies [12]. The integration of SCM-derived EVs analysis into clinical ART workflows might enhance embryo assessment, optimizing outcomes while reducing risks associated with other more invasive testing methods.

## 2. Biogenesis of Extracellular Vesicles

**Figure 1:**
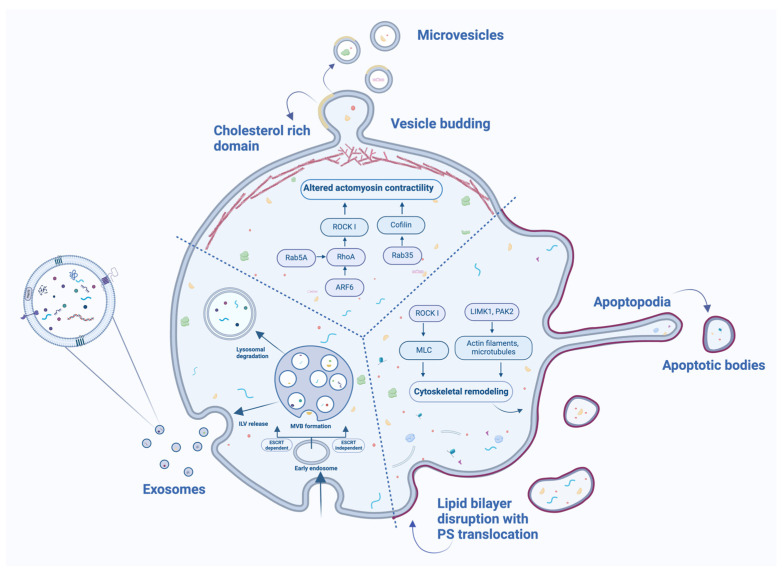
Schematic overview of biogenesis of extracellular vesicles (EVs) and associated regulatory mechanisms. Created in BioRender.

Transmission electron microscopy (TEM) provided foundational insights into EVs biogenesis [14]. Once released, EVs play essential roles in intercellular communication, influencing diverse biological processes such as gene expression, immune modulation, and cellular differentiation, which are critical in reproductive health and disease. The literature often characterizes extracellular vesicles into exosomes, microvesicles, and apoptotic bodies based on their size, biogenesis pathways, and release mechanisms. However, the International Society for Extracellular Vesicles recommends the use of the generic term ‘EV’, as most EV separation methods yield overlapping populations and lack universal markers for specific subtypes [15]. A summary of EVs biogenesis, as reviewed in this study, is presented in Figure 1, offering a depiction that complements the textual overview.

The figure illustrates the formation of three major types of EV: exosomes, microvesicles (MVs), and apoptotic bodies (ApoBDs). Exosomes are formed through the endosomal pathway, where intraluminal vesicles (ILVs) are generated within multivesicular bodies (MVBs) via endosomal sorting complex required for transport (ESCRT)-dependent or ESCRT-independent mechanisms. MVs are produced through outward budding of the plasma membrane, a process regulated by the interplay between small guanosine triphosphatases (GTPases), including Rab5A, Rab35, and ARF6, which impact downstream effectors such as Rho-associated protein kinase I (ROCK I) and cofilin. These pathways collectively regulate actomyosin contractility and drive cytoskeletal remodeling required for MVs budding. ApoBDs release is characterized by extensive cytoskeletal reorganization driven by ROCK I, which phosphorylates and activates myosin light chain (MLC), enhancing actomyosin contractility and facilitating membrane blebbing. This reorganization is further supported by the actions of Lim kinase 1 (LIMK1) and p21-activated kinase 2 (PAK2), which regulate actin filament stabilization and dynamics. Translocation of phosphatidylserine (PS) to the outer leaflet of the lipid bilayer signals ApoBDs recognition and clearance.

### 2.1. Exosomes

In the early eighties, TEM research laid the foundation for the first formal description of exosome biogenesis, revealing their origin and trafficking through the endosomal pathway [14]. Exosome formation involves cargo sorting, multivesicular body formation, transport, and fusion with the plasma membrane [16]. The multivesicular body (MVB) is generated through the formation of numerous intraluminal vesicles within the early endosome [17]. This step can either be dependent on the endosomal sorting complex required for transport (ESCRT) machinery or ESCRT independent [18], since the depletion of ESCRT complexes has been found to cause a modulation in the formation of MVBs but not their complete absence [19].

Predominantly exosomes originate from the ESCRT pathway [20]. During this process, the ESCRT complex is recruited to the site of intraluminal vesicles (ILVs) formation. Specifically, ESCRT-0, consisting of Hrs (hepatocyte growth factor-regulated tyrosine kinase substrate) and STAM1/2 (signal transducing adaptor molecule1/2) subunits, interacts through GAT (GGA and TOM1domain) domains to bind phosphatidylinositol 3-phosphate and ubiquitin, thereby initiating the recruitment of ESCRT-I, which is crucial for the sorting of cargo within multivesicular bodies [21,22]. ESCRT-0 engages ubiquitin through multiple ubiquitin-binding domains (UBDs), each exhibiting distinct and independent affinities rather than functioning co-operatively. The Hrs exhibits a higher affinity for ubiquitin compared to the UIM (ubiquitin-interacting motif) and VHS (Vps27, Hrs, STAM) domains of STAM, and the overall affinity of ESCRT-0 is an average of these individual contributions [23].

Ubiquitination serves as a critical sorting signal that directs cargo into the MVB pathway, where ESCRT-I and ESCRT-II are key mediators. ESCRT-I is recruited from cytoplasm to endosomes through interactions with ESCRT-0 and ubiquitinated cargo [24]. Additionally, ESCRT-I assists in membrane budding, since it connects with ESCRT-II. ESCRT-II is a heterotetrameric complex that interacts with ESCRT-I, phosphatidylinositol 3-phosphate (PI3P), and ubiquitin simultaneously, further anchoring ESCRT-II to the membrane [21]. Additionally, the Y-shaped structure of ESCRT-II is critical for nucleation of Snf (sorting nexin filaments) filaments and initiation of ESCRT-III assembly, which is critical for the final scission of the MVB vesicles [25]. Electron microscopy revealed that Snf forms spiraling filaments on lipid monolayers, which are later transformed into membrane-sculpturing helices that assist in cargo sequestration and eventual formation of ILVs during multivesicular body biogenesis [26]. Helical structures of ESCRT- III complex are remodeled by AAA-type ATPase VSP4 (vacuolar protein sorting-associated protein 4) activity, that drives the disassembly of ESCRT-III subcomponents and final steps of vesicle formation and membrane fission [27].

While the ESCRT machinery is essential for MVB biogenesis, some studies indicate that ESCRT-independent pathways also play a significant role in multivesicular body formation. The literature indicates that the monoubiquitination of cargo proteins can be a sufficient sorting signal for entry into MVBs, indicating that MVB biogenesis may involve compensatory mechanisms to ensure proper endosomal function [28]. Moreover emerging evidence points to the importance of other ESCRT-independent factors for MVB biogenesis, such as RAB31. Active RAB31 is a critical regulator of an ESCRT-independent exosome biogenesis pathway since it drives the formation of ILVs by engaging flotillin proteins in lipid raft microdomains. Additionally, RAB31 contributes to the inactivation of RAB7, thereby preventing the fusion of multivesicular bodies with lysosomes [29]. Though the literature typically distinguishes between ESCRT-dependent and ESCRT-independent mechanisms for the formation of ILVs to enhance comprehension, these pathways probably exhibit interconnections and might work synergistically [30].

Once formed, ILVs within MVBs can take different trafficking routes, leading to distinct cellular outcomes. In this context, ceramide, a lipid produced by nSMase2 (neutral sphingomyelinase 2), plays a crucial role in determining MVB fate by influencing the lipid composition of MVB membranes. This lipid modulation by nSMase2 is critical for either directing MVBs toward lysosomal degradation or promoting their secretion as exosomes [31]. Additionally, ISGylation has been proposed as a potential signal affecting MVBs’ fate. Interferon-Stimulated Gene 15 (ISG15) conjugation results in the aggregation and subsequent lysosomal degradation of the MVB protein TSG101, which effectively impairs exosome release and diverts MVBs from the secretory pathway toward degradation [32]. Contrarily, exosome release is facilitated by proteins belonging to the Rab family and SNARE family proteins. Rab27a and Rab27b both play stimulatory roles in the regulation of exosome secretion by facilitating docking and controlling positioning of MVB at the plasma membrane [33]. In addition to Rab27, the exosome secretion is upregulated by Rab35, which orchestrates the docking and tethering of endosomal vesicles with the plasma membrane [34] and deubiquitination of Rab35 by the USP32 (Ubiquitin-Specific Protease 32) enzyme, which prevented its proteasomal degradation [35]. Beyond the involvement of Rab proteins, it has been shown that SNARE complex mediates the fusion of MVBs with the plasma membrane (PM) and plays an essential role in facilitating exosome release [36].

The release of exosomes into the extracellular space depends on the cell type as well as on external factors such as pH, hypoxia conditions, tumor microenvironment, and cytosolic Ca^2+^ concentration, among others [16,20]. Exosomes, which are eventually released from the cell membrane, are subsequently transported to recipient cells. Although the precise mechanisms governing exosome targeting are not yet fully understood, it is known that once exosomes reach their target cells, they can influence them by directly interacting with surface proteins on the plasma membrane, being internalized through endocytosis, or by fusing directly with the recipient cell’s plasma membrane [37]. Notably, it has been demonstrated that different proteins on exosomes can either promote or restrict their uptake by recipient cells. Specifically, A Disintegrin and Metalloproteinase 17 (ADAM17) present on exosomes interacts with integrin α5β1 on recipient cells, thereby promoting exosome binding and internalization. Conversely, tetraspanin CD9 was found to restrict exosome uptake by reducing the efficiency of this interaction [38]. However, it has been suggested that endocytosis is the primary mechanism for exosome uptake, rather than membrane fusion, as confirmed using octadecyl rhodamine B chloride (R18) labeling [39]. Moreover, phagocytosis is another mechanism of exosome uptake where the uptake of exosomes is dependent on the actin cytoskeleton, PI3K activity, dynamin2, and TIM-4 [40]. Internalization pathways for exosomes also include clathrin-mediated endocytosis, macropinocytosis [41], and lipid raft-mediated endocytosis [42].

Upon internalization by the recipient cell, the exosome embarks on an intracellular trajectory, where it can facilitate the delivery of its molecular cargo (proteins, soluble factors, RNA, miRNA, pathogenic agents, retrovirus-like particles, or prion shuttlers) ultimately modulating various signaling pathways and cellular functions [43].

Alternatively, exosomes can be targeted for degradation within lysosomes, where their components are broken down, limiting their impact on the recipient cell [32].

### 2.2. Microvesicles

Microvesicles (MVs) were first identified as lipid-rich vesicles shed from platelet osmophilic granules [44]. In contrast to the intracellular origin of exosomes, MVs are formed through direct outward budding from the plasma membrane, a process that involves extensive lipid and protein rearrangement. This dynamic restructuring requires the activity of calcium-dependent enzymes, such as scramblases and calpain, which disrupt membrane asymmetry and facilitate the formation and release of MVs [17].

A crucial aspect of MVs biogenesis implicates the rearrangement of actin filaments that generate mechanical forces required for the fission of MVs from the plasma membrane. This process is tightly regulated by small GTPases such as ADP-Ribosylation Factor 6 (ARF6), RhoA, and Rab35 which entail control of actomyosin contraction at the neck of budding MV [45]. Moreover, ARF6, a small Ras GTPase, regulates MV shedding by activating phospholipase D, which subsequently recruits ERK (extracellular signal-regulated kinase) to the plasma membrane. ERK activation triggers myosin light-chain kinase (MLCK), leading to phosphorylation of myosin light chain (MLC), facilitating actomyosin contraction, and ultimately driving membrane fission to release MVs [2].

Actin dynamic is further regulated by Rho GTPases and ROCK (Rho-associated protein kinase). RhoA activates a downstream signaling pathway involving Rho kinase ROCK, which subsequently activates Lim kinase (LIMK). This pathway culminates in the phosphorylation of cofilin, thereby promoting the extension of actin filaments and subsequent MV formation [46]. Additionally, Cdc42, a small GTPase of the Rho family, appears specifically implicated in MV biogenesis via its interaction with IQGAP1 (IQ motif-containing GTPase-activating protein 1), a downstream effector involved in actin cytoskeletal organization. Knockdown of either Cdc42 or IQGAP1 significantly reduces MV shedding while having no impact on the production of small extracellular vesicles (sEVs), suggesting a distinct and targeted role for Cdc42-IQGAP1 signaling in MV formation [47]. Moreover, Rab5a orchestrates the reorganization of the actin cytoskeleton. Its activation is mediated through the phosphorylation of AKT, which follows the activation of Protease Activated Receptor 2 (PAR2) by trypsin. The Rab5a-driven actin polymerization facilitates the budding and release of MVs, highlighting the importance of the trypsin-PAR2-Rab5a axis in promoting MV biogenesis [48].

Despite the critical role of actin cytoskeletal dynamics in MV formation, this process is also highly dependent on plasma membrane lipid composition, particularly the presence of cholesterol-rich lipid rafts. It has been demonstrated that MVs primarily originate from these specialized membrane domains, and their formation is significantly impaired when membrane cholesterol is depleted using methyl-β-cyclodextrin (MβCD) [49]. Furthermore, MV shedding is influenced by calcium elevation that leads to activation of calcium-dependent proteases like calpain and increased exposure of phosphatidylserine on the outer membrane leaflet [50].

Following the completion of MV scission from the plasma membrane, MV uptake by recipient cells can occur through multiple mechanisms, including receptor-mediated uptake and macropinocytosis, indicating that diverse pathways facilitate internalization based on the surface protein composition of both MVs and target cells [51]. Notably, MVs are thought to predominantly utilize SNARE-mediated membrane fusion to deliver cargo directly to the recipient cells, thereby bypassing endocytic degradation pathways and enhancing delivery efficiency [52].

### 2.3. Apoptotic Bodies

Apoptotic bodies (ApoBDs), a subtype of EVs, are generated exclusively during the final stages of apoptosis, a process regulated by the Bcl-2 protein family [53]. They are typically larger than other EV populations, as they encapsulate diverse cellular components [51]. They are characterized by cytoskeletal and membrane alterations, including phosphatidylserine (PS) translocation to the outer lipid bilayer [54].

Similarly to the release of MVs, the biogenesis of ApoBDs also requires cytoskeletal rearrangements, as demonstrated through treatment with microfilament-disrupting agents like cytochalasin B [55]. Actomyosin contraction and membrane dynamics during ApoBDs formation are regulated by key signaling proteins such as ROCK I [56]. ROCK I is cleaved by caspase-3 during the execution phase of apoptosis, resulting in the removal of its C-terminal inhibitory domain and constitutive activation. This active form of ROCK I drives MLC phosphorylation through direct phosphorylation of MLC or by inhibition of MLC phosphatase, generating the actin-myosin contractile force necessary for membrane blebbing—a hallmark of apoptosis [57]. Studies that utilized the ROCK inhibitor Y-27632 to assess the dependency of membrane blebbing on ROCK I activity demonstrated that inhibition of ROCK I significantly reduces membrane blebbing [58].

Additionally, caspase-3 activates a complementary pathway by cleaving LIMK1, which phosphorylates cofilin, leading to actin filament stabilization and reorganization [59]. PAK2 (p21-activated kinase 2) is another regulatory kinase that functions as an effector for the small GTPases Rac and Cdc42, playing a role in the regulation of cytoskeletal remodeling by phosphorylating proteins that control actin and microtubule dynamics [60]. Despite the contributions of LIMK1 and PAK2 to cytoskeletal dynamics, knockout studies identify ROCK I as the critical, non-redundant regulator of apoptotic membrane blebbing and cell disassembly, while LIMK1 and PAK2 presumably have minimal roles in these processes [61].

While membrane blebbing is commonly considered the primary pathway for ApoBD formation, diverse cell types may manifest various forms of membrane deformation during apoptosis. These can include microtubule spikes, apoptopodia, or beaded apoptopodia [53]. Regardless of actin filaments playing a significant role in membrane blebbing, they may not be essential for the actual fragmentation of ApoBDs, as inhibitors like cytochalasin D did not completely abolish ApoBD formation. Moreover, it has been established that microtubules and intermediate filaments, particularly vimentin, contribute to ApoBD formation by stabilizing apoptotic protrusions [62]. This highlights the complexity of ApoBDs and suggests that different subsets of ApoBDs could serve distinct physiological roles [63].

After the completion of biogenesis, ApoBDs are released into the extracellular environment, where they can be recognized and engulfed by phagocytes [64]. Their improper clearance can lead to autoimmune diseases, while their ability to carry bioactive molecules, such as antigens, DNA, or cytokines, influences inflammation, tissue repair, and immune modulation, underscoring their complex roles in both physiological and pathological processes [65].

## 3. Extracellular Vesicles from Animal Embryo Spent Culture Media

Overview of studies investigating extracellular vesicles from animal embryo spent culture media, conducted across diverse animal models is summarized in Table 1.

Recent research on EVs from spent embryo culture medium has highlighted significant associations between EV secretion profiles and embryo quality, suggesting their utility as biomarkers of developmental competence. Foundational work demonstrated that embryos with lower developmental potential secreted higher EV concentrations compared to viable embryos, likely reflecting stress-induced EV release. This relationship has been consistently observed across studies, with findings indicating that elevated EVs’ production signals developmental stress, whereas a more controlled, homogeneous EV profile may reflect cellular stability and higher bovine embryo quality [66,67,68,69]. Further studies into EVs’ characteristics have shown that viable early-blastulating bovine embryos tend to secrete larger EVs, reinforcing the relationship between embryo competence and EVs phenotype [67,68].

However, findings vary regarding the interpretation of EV size as a developmental marker. While many studies associate larger EVs with viable embryos, other research reports that non-viable embryos produce larger, more abundant EVs than their viable counterparts, suggesting EVs size may not consistently reflect bovine embryo competence [69]. Additionally, studies indicate that both the concentration and size of EVs are greater in blastocyst-derived embryos, potentially reflecting increased EV production in developmentally competent embryos [70]. Research further shows that embryos release higher EV concentrations during later developmental stages, likely due to increased cellular activity and complexity as they progress through stages such as blastulation [70].

The molecular cargo within EVs, particularly miRNAs, has emerged as a promising biomarker for embryo viability. The extensive miRNA profiling of EVs has identified specific miRNA signatures unique to viable versus non-viable embryos, establishing miRNA content as a non-invasive marker for bovine embryo quality [66]. Further studies identified distinct miRNA expression profiles between bovine embryos arrested in development and those progressing to the blastocyst stage [69,71]. Specifically, miR-103, miR-100, miR-502a, and miR-1 were upregulated in arrested embryos, whereas miR-92a, miR-140, miR-2285av, and miR-222 were downregulated. KEGG pathway enrichment analysis linked upregulated miRNAs in arrested embryos to pathways in fatty acid biosynthesis, lysine degradation, and pluripotency regulation. These findings suggest that differential miRNA expression plays a critical role in embryo competence by modulating key processes like fatty acid metabolism, lysine degradation, and gap junction signaling, supporting the potential of miRNA profiling as a non-invasive tool for assessing embryo viability [69]. In examining miRNA profiles throughout different developmental stages, compaction-stage embryos were found to upregulate miRNAs involved in oxytocin and estrogen signaling, while blastulation-stage EVs contained miRNAs were linked to prolactin signaling—both essential for embryo–endometrium interaction. Specific miRNAs, such as miR-21 and miR-130, appear across both stages, likely playing roles in embryonic genome activation and the maternal-to-embryonic transition. Meanwhile, miR-30c, associated with lower embryo quality and apoptosis, appears specifically during compaction, reflecting EV cargo’s dynamic nature and its alignment with the bovine embryo state [70]. Although these findings highlight the biomarker potential of EVs miRNA signatures, inconsistencies in miRNA isolation methods and sequencing highlight the critical need for standardized protocols to enable cross-study validation.

Expanding beyond miRNAs, researchers have investigated DNA content within EVs as a potential diagnostic tool. Observations of minimal DNA content differences between ApoBDs and nanovesicles (nEVs) suggest non-selective DNA packaging within EVs, while findings indicate DNA presence in EVs from both high-quality blastocysts and arrested embryos, independent of apoptotic rates as demonstrated on mouse and bovine embryos [72,73]. These observations suggest that EVs could serve as a reliable, non-invasive genetic diagnostic resource without requiring biopsy, especially since viable embryos with low apoptotic rates also release DNA-containing EVs suitable for genotyping. Further research is needed to determine whether certain DNA sequences are selectively packaged in EVs, which could greatly enhance the utility of EV DNA for diagnostics in assisted reproductive technologies.

Culture conditions, particularly oxygen tension and media renewal protocols, significantly influence EV composition and functionality. Studies indicate that oxygen level variations can alter production, concentration, and miRNA profiles within EVs. Differential expression of miRNAs under altered oxygen tension, especially those involved in signaling pathways like Forkhead Box O (FOXO), p53, and Transforming Growth Factor-Beta (TGF-beta), suggests that EVs can regulate pathways related to cell proliferation and stress response. Elevated miR-210 levels under low oxygen tension align with its role as a hypoxia marker, suggesting miR-210 could serve as a potential non-invasive biomarker for monitoring oxygen conditions in bovine embryo culture media [74]. Comparative analyses of in vitro and in vivo culturing conditions for bovine embryos reveal that in vitro embryos produce a higher concentration of sEVs with a distinct miRNA profile, while EVs from in vivo embryos are enriched in miRNAs targeting key developmental pathways such as Ras, MAPK, oxytocin metabolism, and glycerophospholipid metabolism. In vitro-derived EVs were associated with pathways like lysine degradation, HIF-1, and WNT signaling, reflecting cellular stress and adaptation responses [75]. Such results underscore the necessity of optimizing in vitro culture conditions to better replicate in vivo environments, thereby enhancing EV functionality and embryo viability in ART.

Further research indicated that EVs from in vitro bovine embryos uniquely activate non-classical interferon-stimulated genes in endometrial cells [76]. Moreover, EVs secreted in vitro notably upregulate gene expression related to cell signaling, including WNT7A and oxytocin receptor (OXTR), an effect not observed in EVs from in vivo embryos. This highlights the differential signaling influence of EVs based on embryo origin and underscores the impact of culture conditions on EV signaling capabilities [77].

The functional role of EVs extends beyond their use as biomarkers, as studies conducted on bovine and porcine embryo conditioned culture indicate that EVs facilitate embryo-embryo communication necessary for synchronized development. In particular, the depletion of EVs from culture media has been shown to impair blastocyst formation rates, reflecting EVs critical roles in developmental progression [68]. Moreover, embryo-derived exosomes are crucial for intercellular signaling and embryo developmental competence enhancement, as experiments indicate that nonrenewal culture conditions preserve exosome concentrations in spent media, resulting in higher blastocyst formation rates and improved embryo quality metrics compared to renewal conditions, which likely deplete beneficial EVs. Exosome supplementation in renewed media partially rescued these impairments, enhancing blastocyst formation, total cell count, intracellular mass/trophectoderm ratios, and pluripotency via increased *Oct-4* expression [78]. The enhanced expression of pluripotency-related mRNAs like Oct4, Klf4, and NANOG has previously been observed in EVs secreted by parthenogenic embryos. Focusing on the dynamics of EV-mediated communication, the research found that the continuous presence of EVs during co-culture improved embryonic development more effectively than periodic supplementation, underlining the need for sustained EV availability in supporting developmental processes [79].

To deepen our understanding of the molecular contributions of EVs, the PIBF (Progesterone-Induced Blocking Factor)-containing EVs produced by mouse embryos promote an immune environment supportive of pregnancy by enhancing anti-inflammatory cytokine IL-10 production [80]. Moreover, EVs were shown to alter transcriptomic profiles of endometrial cells, upregulating interferon-related genes in response to pre-hatching bovine embryo EVs [76]. However, limitations in protein quantification suggest that immune-regulatory molecules within EVs may be underrepresented in current findings, pointing to a need for advanced proteomic methods to verify these effects.

Furthermore, functional studies confirmed the internalization of labeled EVs by recipient porcine and bovine embryos, highlighting their role in paracrine signaling and inter-embryo communication [79,81]. Proteomic and RNA sequencing of sheep embryo-derived EVs further revealed that EVs from the culture media contained 231 proteins and 512 mRNAs. These EVs were found to accumulate in the uterine epithelial cells, supporting the idea that the conceptus secretes EVs to communicate with the maternal environment. These findings reveal a bi-directional communication mechanism, where EVs from both the conceptus and the uterus contribute to the complex signaling processes essential for pregnancy establishment without penetrating deeper maternal tissues [82].

In the realm of ART, EVs hold therapeutic potential since embryo-derived EVs may mediate embryo-embryo communication and enhance collective embryo health. Research undertaken on bovine and mouse embryos demonstrated that supplementing culture media with EVs or specific miRNAs from EVs, like miR-378a-3p, significantly enhances blastocyst quality, hatching rates, and implantation potential, underscoring the value of EVs in optimizing culture conditions for developmental success [71,81,83]. Interestingly, bovine embryos in EV-depleted media show altered gene expression linked to pluripotency, such as reduced *NANOG* and increased *SOX2*, suggesting that EVs may impact embryo gene regulation [84]. High-throughput RNA profiling, such as circRNA and tRNA-derived fragment (tDR) analysis, could provide more detailed insights into EV-mediated regulatory mechanisms. For example, inhibition of tDR-14:32-Glu-CTC-1, a tRNA fragment in EVs, has been shown to increase bovine embryo hatching rates, revealing the functional versatility of EV-derived non-coding RNAs [85]. Such therapeutic application highlights EVs potential value as intrinsic regulators of embryonic competence through sustained inter-embryo signaling. Future research will likely emphasize refining EVs’ enrichment and profiling methods to enhance purity and functional clarity.

The presence of DNA in EVs has also gained attention as a potential marker for genetic screening without biopsy. Minimal DNA differences were revealed between ApoBDs and nanovesicles (nEVs) derived from mouse embryos, implying non-selective DNA packaging [72]. Similar findings indicate that both high-quality and arrested bovine embryos released DNA-positive EVs, suggesting DNA content is an independent marker of apoptotic activity. Therefore EV-derived DNA could offer a reliable tool for genetic screening without the need for biopsy [73]. The need for further research is emphasized by evidence that some DNA may be attached to the external surface of EVs, as demonstrated by changes in clustering patterns in sequencing data observed before and after DNase treatment of certain EV fractions [72]. Validation of EV-DNA is necessary to ensure compatibility with traditional diagnostic methods, potentially expanding the diagnostic capabilities of EVs in ART.

To advance therapeutic applications of embryo-derived EVs in ART, it will be essential to implement improved characterization methods and establish unified protocols for EV enrichment, ensuring accurate validation of EV identity and content. Such standardized approaches will enhance the reproducibility and reliability of EV-based interventions, ultimately contributing to more effective and targeted outcomes.

**Table 1:** Extracellular vesicles (EVs) in spent animal embryo culture media: an overview of isolation and characterization techniques, and their role in embryo quality assessment.

Reference	Model	Methods for EV Enrichment	Methods for EVs Characterization	Main Findings
[66]	Bovine embryos	Centrifugation	NTA, TEM, Flow Cytometry with markers CD9, CD63	EVs secreted by blastocysts may serve as indicators of developmental competence, with their concentration and characteristics differing (not statistically) based on embryo origin and competence.
[67]	Bovine embryos	Centrifugation	NTA, TEM, Flow Cytometry for markers CD9, CD63, CD81; RNA sequencing	EV characteristics (size, concentration) and miRNA content varied with embryo viability, indicating that EV profiles may serve as non-invasive markers for embryo quality assessment during blastulation.
[68]	Bovine embryos	Sequential centrifugation, size exclusion chromatography	NTA, TEM, SEM, EV array	Higher EV concentration and smaller EV sizes were linked with embryos of lower developmental potential; EV profiles correlated with embryo quality, suggesting their potential as non-invasive quality markers. The depletion of EVs from culture media results in lower blastocyst formation rate.
[69]	Bovine embryos	Centrifugation	NTA, TEM, Flow Cytometry with surface markers CD9, CD63, CD81, CD40, miRNA content analysis	Embryos that reach advanced developmental stage secrete lower amounts of EVs compared to arrested embryos. 8 miRNA were differentially expressed between EVs secreted from arrested embryos and embryos that reached blastocyst stage.
[70]	Bovine embryos	Centrifugation	NTA, TEM, Flow Cytometry for surface markers CD9, CD63, CD81, miRNA sequencing	EV concentration increased from compaction to blastulation, with distinct miRNA profiles between stages. Differentially expressed miRNAs were linked with pathways essential for early development, suggesting EVs play a role in embryo-maternal communication.
[71]	Bovine embryos	Centrifugation	NTA, TEM, Western Blot (CD 63, CD9, TSG101, ApoA-I, AGO-2), miRNA content analysis	EV characteristics—morphology, concentration, and markers—had discriminative value since blastocysts secreted higher concentrations of larger EVs enriched in markers CD9 and TSG101 compared to the non-blastocyst samples. RNA-378a-3p up-regulated in EVs from blastocysts promoted embryo hatching and improved embryo quality.
[72]	Mouse embryos	Differential ultracentrifugation	NTA, TEM, immunogold labeling against CD63 and ARF6, DNA staining	EVs contained DNA sequences enriched for genes linked to embryonic origin. Their DNA profiles reflected the whole genome, showing no differences between apoptotic bodies and other EV subtypes, suggesting DNA release is independent of apoptosis.
[73]	Bovine embryos	Ultrafiltration	NTA, TEM, Western Blot (TSG101, CD9, APOA1,ALIX), DNA assay kit	DNA content in EVs was independent of the apoptotic rate.
[74]	Bovine embryos	Precipitation, centrifugation	NTA, TEM, Western Blot (CD 63 and GRP78), miRNA content analysis	EV concentration was higher under high oxygen tension on day 7 but lower on day 3. Distinct miRNA profiles under different oxygen levels suggest oxygen tension affects EV-mediated communication related to embryo development.
[75]	Bovine embryos	Precipitation, centrifugation	NTA, TEM, RT-qPCR for miRNA profiling of EVs	In vivo embryos secreted fewer EVs than in vitro, with distinct miRNA profiles between the two groups. The different miRNAs in EVs are predicted to influence pathways critical for embryo-maternal communication and pregnancy establishment.
[76]	Bovine embryos	Differential ultracentrifugation	NTA, TEM, Flow Cytometry for surface markers CD9, CD63, CD81, and CD40, labeling with PKH67 fluorescent dye to assess internalization	EVs from in vivo and in vitro embryos were internalized by endometrial cells, activating IFNT-stimulated genes. In vitro-derived EVs induced a greater number of differentially expressed genes, suggesting that embryo origin influences EV-mediated endometrial responses.
[77]	Bovine embryos	Centrifugation and size exclusion chromatography	NTA, TEM, Flow Cytometry for surface markers CD9, CD63, CD81, and CD40; PKH67 labeling to assess EV internalization	EVs from in vitro-produced embryos upregulated endometrial genes related to IFN signaling, including *ISG*s and *OXTR*.
[78]	Bovine embryos	Differential centrifugation	TEM, fluorescence microscopy combined with CD9 immunostaining	Blastocyst formation rate was significantly enhanced by exosome supplementation, the inner cell mass to trophectoderm ratio was increased, calving rates post-transfer were improved, and expression of *Oct-4* was increased.
[79]	Porcine embryos	Differential centrifugation	TEM, immunofluorescence for CD9 marker, mRNA profiling. Internalization tracked by PKH67 dye and confocal microscopy.	Extracellular vesicles secreted by embryos contained pluripotency related mRNAs and were internalized by cloned embryos, enhancing their cleavage rates, blastocyst formation, and pluripotency gene expression.
[80]	Mouse embryos	Differential centrifugation	TEM, Immuno-Electron Microscopy, Flow Cytometry with Annexin V and IL-10 markers	PIBF-positive EVs from embryos bound to CD8+ T cells, increasing IL-10 production, which supports immune tolerance. Blocking PIBF on EVs reduced this effect, indicating PIBF’s role in embryo-immune communication during early pregnancy.
[81]	Bovine embryos	Density gradient ultracentrifugation	NTA, TEM, immunogold labeling against CD63, Western Blot (CD63, CD9, TSG101, Ago-2, ApoA-I), confocal microscopy with PKH67 labeling	EVs were shown to be internalized by other embryos, showing potential as embryotropins for improving embryo quality and development. EVs supplementation of culture media resulted in improved blastocyst rate and lower apoptotic cell ratio.
[82]	Sheep embryos	Centrifugation, filtration	NTA, TEM, PKH67 labeling, HPLC-MS/MS for proteomics, RNA sequencing	Embryo derived EVs carried proteins and mRNAs that support embryo-maternal signaling, localizing specifically to uterine luminal epithelium, which suggests a role in early pregnancy communication.
[83]	Mouse embryos	Centrifugation	TEM, Western Blot for CD9 and TSG101, Confocal Microscopy (Exo-Green/Exo-Red labeling)	EVs from outgrowth embryos enhanced blastocyst formation, increased total cell number, and reduced apoptosis. Post-implantation, EV-supplemented embryos showed improved implantation rates, indicating a supportive role in early embryonic development and implantation potential.
[84]	Bovine embryos	Ultrafiltration	NTA, TEM, Flow Cytometry with markers CD9, CD63, CD81, CD40L. Internalization tracked by PKH67 dye and fluorescence microscopy	Nanoparticles (NP) from the culture medium were internalized by embryos at all stages, with depletion affecting morphological quality of blastocysts and *SOX2* and *NANOG* expression, suggesting an influence of NP on embryo functionality.

## 4. Extracellular Vesicles from Human Embryo Spent Culture Media

An overview of studies investigating extracellular vesicles from human embryo spent culture media is summarized in Table 2.

Pioneering studies in 2017 explored the potential of EVs in human embryo culture media as non-invasive markers for embryo viability. Fundamental research used transmission electron microscopy and flow cytometry to confirm the presence of nucleic acid-containing EVs, identifying a critical correlation between EVs characteristics and implantation potential. Flow cytometric analysis revealed that embryos likely to result in successful pregnancies exhibited significantly lower counts of propidium iodide-positive (PI+) EVs, highlighting a link between reduced nucleic acid content in EVs and higher developmental competence. Receiver operating characteristic analysis established a PI+ EV count threshold, with an area under the curve of 0.91, underscoring the assay’s diagnostic accuracy for predicting implantation success. This innovative, non-invasive approach demonstrated that EVs, particularly those marked by nucleic acid content, offer an alternative to traditional embryo selection methods, advancing the clinical application of EVs as biomarkers in assisted reproductive technology [86].

The potential for EVs in spent culture to serve as indicators of embryo viability was further validated through a comprehensive analysis of miRNA expression patterns and EV concentrations in spent embryo culture media, elucidating their relationship with pregnancy outcomes. Using a high-throughput microarray approach, 621 distinct miRNAs were identified in SCM, with embryos leading to successful pregnancies exhibiting notably fewer miRNAs on average compared to those with failed outcomes. Nanoparticle tracking analysis (NTA) confirmed size diversity in EVs, suggesting functional variability. Lower EV concentrations were observed in embryos that were successfully implanted compared to non-viable embryos, suggesting that diminished EV secretion is a potential marker of embryo competence. However, a larger dataset would be required to clarify the specific roles of EVs in embryo implantation and pregnancy success. Functional pathway analysis further highlighted enrichment in miRNAs associated with negative pregnancy outcomes in pathways linked to IL-6 signaling, cell cycle regulation, and epithelial cell proliferation—processes integral to implantation and early development [87].

In a larger sample cohort, researchers identified significant patterns in embryo-derived EVs. Using TEM and NTA, the research identified a predominantly exosome-like population of EVs, typically under 100 nm, with increased concentrations noted at the blastocyst stage. CD9 and CD63 tetraspanins, confirmed through immunogold labeling and Western blotting, validated the exosomal nature of these vesicles. Furthermore, fluorescence microscopy demonstrated the successful internalization of labeled EVs by maternal endometrial cells, particularly those derived from day 5 blastocysts, highlighting a potential mechanism for early embryo-endometrium communication critical to implantation. EVs containing pluripotency-related transcripts (e.g., POU5F1, NANOG) and immune-regulatory proteins (e.g., HLA-G) may actively promote a receptive endometrial environment [88].

Evidence of EV release and embryo developmental stage correlation was extended by electron microscopy analysis of human embryos from zygote to blastocyst stages that revealed the consistent release of CD9-positive EVs across preimplantation development, with vesicles traversing the zona pellucida into the perivitelline space and culture media. Immunogold labeling of CD9 confirmed the exosomal nature of these vesicles, which average size between 50–200 nm, aligning with typical exosome dimensions. TEM showed that these EVs permeate the ZP, indicating that embryonic EVs could facilitate a direct signaling pathway between embryo and maternal tissues. Notably, blastocyst-stage embryos secreted a greater concentration of EVs, suggesting an increased demand for maternal communication as the embryo prepares for implantation [89]. However, relying solely on CD9 risks omitting non-tetraspanin-expressing EV populations, necessitating more inclusive markers for comprehensive analysis [90].

Further supporting the presence of EVs in human embryo culture media, another study confirmed EVs content. Analysis of EVs from conditioned media associated with successful pregnancies revealed distinct differences in size, concentration, and miRNA content compared to EVs from non-implanting and degenerated embryos. Characterized through NTA, TEM, and Western blotting for EV-specific markers CD9 and CD63, EVs derived from SCM of implanted embryos exhibited a higher particle concentration and larger mean diameter relative to EVs derived from SCM of non-implanted embryos, indicating that EVs characteristics in conditioned media may reflect embryo quality and implantation potential. While sample pooling limits the ability to analyze EVs profiles at the individual blastocyst level, these findings underscore the potential of EVs in conditioned media as biomarkers in IVF [91].

Exploring genomic DNA (gDNA) within EVs, additional insights were provided regarding EVs heterogeneity and their potential as a biomarker for embryo quality and chromosomal integrity in human embryos. Through NTA and array comparative genomic hybridization (aCGH), the researchers found a distinct correlation between EVs size and embryo quality, with larger EVs associated with higher-quality embryos. Chromosomal abnormalities were prevalent in EV-derived gDNA, especially in arrested embryos. The abnormality rate in EVs was notably higher than in embryos, suggesting potential DNA contamination and underscoring the need for further studies to assess the feasibility of using EV-derived gDNA for embryo ploidy prediction [13]. Moreover, complementing protein markers with lipid markers, as recommended by ISEV, would strengthen the research findings [15].

Finally, in 2023, findings linked EV count in culture media with blastocyst morphology, though low centrifugal force likely introduced heterogeneity within the sample. This study pioneered in quantification of placental alkaline phosphatase (PALP)-positive EVs, introducing a potential new marker for assessing embryo competence [92].

Recent findings reveal that EVs released by aneuploid embryos possess distinct transcriptomic profiles, with unique RNA markers such as upregulated *TMED10* (transmembrane Emp24 domain-containing protein 10) and downregulated *PPM1J* (protein phosphatase, Mg^2+^/Mn^2+^ dependent 1J) and *LINC00561*, indicating potential non-invasive indicators of aneuploidy. Exposure of decidualized endometrial stromal cells (dESCs) to these EVs lead to upregulated *MUC1* expression—a known barrier to embryo implantation, suggesting a mechanism by which aneuploid embryos may actively inhibit endometrial receptivity, thus decreasing implantation potential. Enrichment analysis further revealed that aneuploid EVs affect pathways related to cellular communication and apoptosis, contrasting with euploid EVs, which support genes promoting cytokine interactions and steroid biosynthesis, both favorable to implantation. Such findings highlight the utility of EV-derived RNA profiles as non-invasive biomarkers for detecting aneuploidy, offering a promising alternative to conventional preimplantation genetic testing [12].

Collectively, these studies affirm the presence of EVs in human embryo culture media and point to their potential role in embryo viability and embryo-maternal signaling. Future research should integrate an orthogonal-method approach in EVs isolation and characterization to differentiate between EVs and other extracellular nanosized particles accurately [93].

**Table 2:** Extracellular vesicles (EVs) in spent human embryo culture media: an overview of isolation and characterization techniques, and their role in embryo quality assessment.

Reference	Model	Methods for EVs Enrichment	Methods for EVs Characterization	Main Findings
[12]	Human embryo, IVF	Differential ultracentrifugation, ultracentrifugation	NTA, TEM, RNA sequencing	EVs from aneuploid embryos showed a distinct transcriptomic profile with specific RNAs (e.g., PPM1J, LINC00561, TMED10) enriched. Aneuploid EVs upregulated MUC1 expression in decidualized endometrial cells, potentially signaling embryo quality to the endometrium.
[13]	Human embryo, ICSI	Differential centrifugation and ultrafiltration	NTA, TEM, Flow Cytometry for surface markers CD9, CD63, CD81, and CD40L; aCGH for gDNA content analysis	Larger EVs were associated with higher embryo quality. gDNA was detected in EVs with a high rate of chromosomal abnormality.
[91]	Human embryo, IVF	Size exclusion chromatography	NTA, TEM, Western Blot for markers CD9 and CD63; RT-qPCR	EVs from implanted and non-implanted embryos showed differences in size, concentration, and miRNA content. Implanted embryo EVs had a higher particle concentration and size, indicating potential for EVs as non-invasive markers for implantation potential.
[86]	Human embryo, IVF	Centrifugation	TEM and Flow Cytometry with Annexin V and PI labeling	PI+ EV counts were significantly lower in media from embryos that successfully implanted. Lower PI+ EV counts in conditioned medium indicate a potential non-invasive marker for identifying viable embryos prior to transfer.
[87]	Human embryo, IVF	Centrifugation	NTA, TEM, Microarray and RT-qPCR	Higher EV concentrations were observed in SCM associated with failed pregnancies compared to successful ones. Certain miRNAs, such as miR-29c-3p and miR-634, were identified as potential biomarkers for predicting pregnancy outcomes.
[88]	Human embryo, IVF	Ultracentrifugation	NTA, TEM with immunogold labeling for CD9 and CD63; Western Blot for exosome markers CD9, CD63, and ALIX; Flow Cytometry	EVs from embryos contained pluripotency-related mRNAs (e.g., NANOG, POU5F1) and HLA-G protein, with higher HLA-G levels in blastocyst-stage EVs. Dye-labeled EVs were visualized being taken up by endometrial cells, suggesting a mechanism for embryo-maternal communication.
[89]	Human embryo, IVF	Ultracentrifugation	NTA, TEM, SEM, immunogold labeling with CD9	CD9-positive EVs were released from embryos at all developmental stages, and these EVs crossed the zona pellucida into the surrounding culture medium. EV concentration was higher in media from blastocysts, suggesting an increase in EV secretion during advanced embryonic stages.

## 5. Methods

This review systematically analyzed the existing literature on extracellular vesicles (EVs) enriched from spent embryo culture medium. We conducted a literature search across databases PubMed, Google Scholar, and ScienceDirect to identify existing research published in English, from 1960 through June 2024. The search utilized a combination of relevant keywords to capture a wide range of studies on EVs within spent embryo culture media. The primary keywords included: “exosomes”, “human embryo”, “spent media”, “blastocyst”, “extracellular vesicles”, “apoptotic bodies”, “microvesicles”, “culture medium”, and “embryo”. Boolean operators (AND, OR) were employed to refine search results and ensure comprehensive retrieval of articles, such as using combinations like “extracellular vesicles AND spent media AND blastocyst” or “microvesicles OR exosomes AND embryo culture medium”. Studies were selected based on the inclusion criteria involving literature published in English and research involving analysis of EVs enriched from spent embryo culture medium, covering either human or animal models. Exclusion criteria were applied to filter out articles not focusing on EVs from spent embryo culture media.

## 6. Current and Future Research Perspectives

The existing literature on extracellular vesicles (EVs) secreted by embryos in spent culture media provides valuable insights into embryonic development, non-invasive diagnostics, and intercellular communication. EVs demonstrate notable variability in both quantity and composition throughout embryo development [69,70,89], with EV concentration, size, and molecular cargo emerging as potential indicators of developmental competence [13,66,67,68,69,71,91]. Embryos appear to utilize EVs for stage-specific signaling [70,84], with shifts in EV composition closely reflecting the embryo’s developmental stage and overall viability, [70,71,91]. The role of culture conditions in EV production has proven critical, with factors such as oxygen tension shown to directly influence EV release, quality, and molecular cargo [74,75,76]. Adjusting these parameters could enhance embryo quality assessments and improve ART outcomes by optimizing the embryonic microenvironment to foster development. Regulatory functions of different RNAs within EVs, such as mRNA, miRNAs, and tRNA-derived fragments, may contribute to embryo quality regulation, hatching, and maternal receptivity, thereby playing a pivotal role in embryo-maternal communication during preimplantation development [12,67,69,70,71,74,75,82,84,85,87,88,91]. Moreover, the potential of EV-derived DNA as a non-invasive genetic screening tool opens promising avenues to reduce the need for embryo biopsies in ART [13,73]. Moreover, the enrichment of embryo culture media with EVs carrying regulatory molecular cargo presents a promising approach to refine ART practices, as these vesicles may enhance embryo development and communication within the maternal environment, ultimately supporting improved implantation outcomes [68,77,78,81,83,84,91].

However, methodological discrepancies, such as differences in EV isolation protocols and inconsistent molecular characterization, pose challenges, limiting comparability across studies and constraining the generalizability of findings. Variability in EVs enrichment techniques additionally impacts EVs purity, yield, and population heterogeneity, emphasizing the need for the implementation of standardized methods [15]. Without uniform EV-specific markers and characterization protocols, direct comparisons are hindered, underscoring the importance of methodological consistency to fully harness EVs as biomarkers or therapeutic agents in ART. Future research should prioritize addressing these methodological challenges through refined EV enrichment, purification, and molecular profiling techniques. Implementing additional methodologies, such as atomic force microscopy, enzyme-linked immunosorbent assay (ELISA), and advanced flow cytometry (FC) (nano FC), offer the potential to enhance the characterization of EVs, providing deeper insights into their biophysical properties and molecular profiles [94,95,96]. Furthermore, in vivo validations and functional assays in recipient maternal tissues are essential to confirm the diagnostic and therapeutic value of EVs in reproductive biology.

## 7. Conclusions

In conclusion, EVs in spent embryo culture media hold substantial potential as biomarkers and mediators of essential early embryo developmental processes, presenting a transformative opportunity in reproductive science. Further investigation into the molecular profiles, size, concentration characteristics, and regulatory mechanisms of EVs, alongside the impact of culture media conditions on their biology, holds the potential to revolutionize embryo assessment, enhance embryo selection, and improve pregnancy outcomes in ART, ultimately deepening our understanding of embryo-maternal interactions, and positioning EVs as a pivotal tool in advancing the future of reproductive medicine.
